# Repeat stent implementation for recanalization of the proximal right coronary artery: a case report

**DOI:** 10.1186/s13256-018-1897-3

**Published:** 2018-12-30

**Authors:** Yabin Wang, Lei Gao, Ming Zhang, Yundai Chen, Feng Cao

**Affiliations:** 1 Department of Cardiology & National Clinical Research Center for Geriatric Diseases, Chinese PLA General Hospital, Beijing, 100853 China; 2 Department of Cardiology, Chinese PLA General Hospital, Beijing, 100853 China

**Keywords:** Stent occlusion, False lumen, Intravascular ultrasound, Percutaneous coronary intervention

## Abstract

**Background:**

A stent in a false lumen is a common cause of stent occlusion after coronary percutaneous coronary artery intervention therapy, particularly in the culprit lesion of acute myocardial infarction. Here, we present an unusual case of successful recanalization of the proximal right coronary artery with implementation of another stent to crush the previous stent in the false lumen.

**Case presentation:**

A 40-year-old Chinese man underwent coronary stent implementation in the proximal right coronary artery due to acute inferior wall myocardial infarction at another hospital. Six months later, he underwent coronary angiography re-examination for recurrent symptomatic angina at our hospital. Coronary angiography and intravascular ultrasound confirmed that the previous stent was deployed in the false lumen of the right coronary artery. Then, intravascular ultrasound was used to guide the wire to re-enter the true lumen of the proximal right coronary artery, and another stent was deployed into the true lumen to crush the previous stent.

**Conclusion:**

Intravascular ultrasound proved to be a pivotal tool in confirming false or true lumen, as well as determining favorable proximal site entry points to avoid rewiring the mesh of the previous stent.

## Introduction

A stent in a false lumen is a common cause of stent occlusion after coronary percutaneous coronary artery intervention (PCI) therapy. In particular, in the culprit lesion of acute myocardial infarction (AMI), it is easier for the wire to go through the false or dissection lumen [1]. Repercutaneous intervention treatment, including wiring the true lumen and exclusion stenting of the dissection flap, is usually performed. Detailed descriptions of techniques for guiding the wire to re-enter the true lumen with chronic total occlusions have been published. Here, we present an interesting case of successful intentional false lumen stenting with re-entry into the true lumen of the proximal right coronary artery (RCA) and implementation of another stent to restore Thrombolysis In Myocardial Infarction (TIMI) grade III coronary flow.

In this case, the procedural details of how to open the false lumen, perform intravascular ultrasound (IVUS)-guided wire into the true lumen, and avoid rewiring the mesh of the previous stent, which differed from other cases, were described in detail.

## Case presentation

**Fig. 1:**
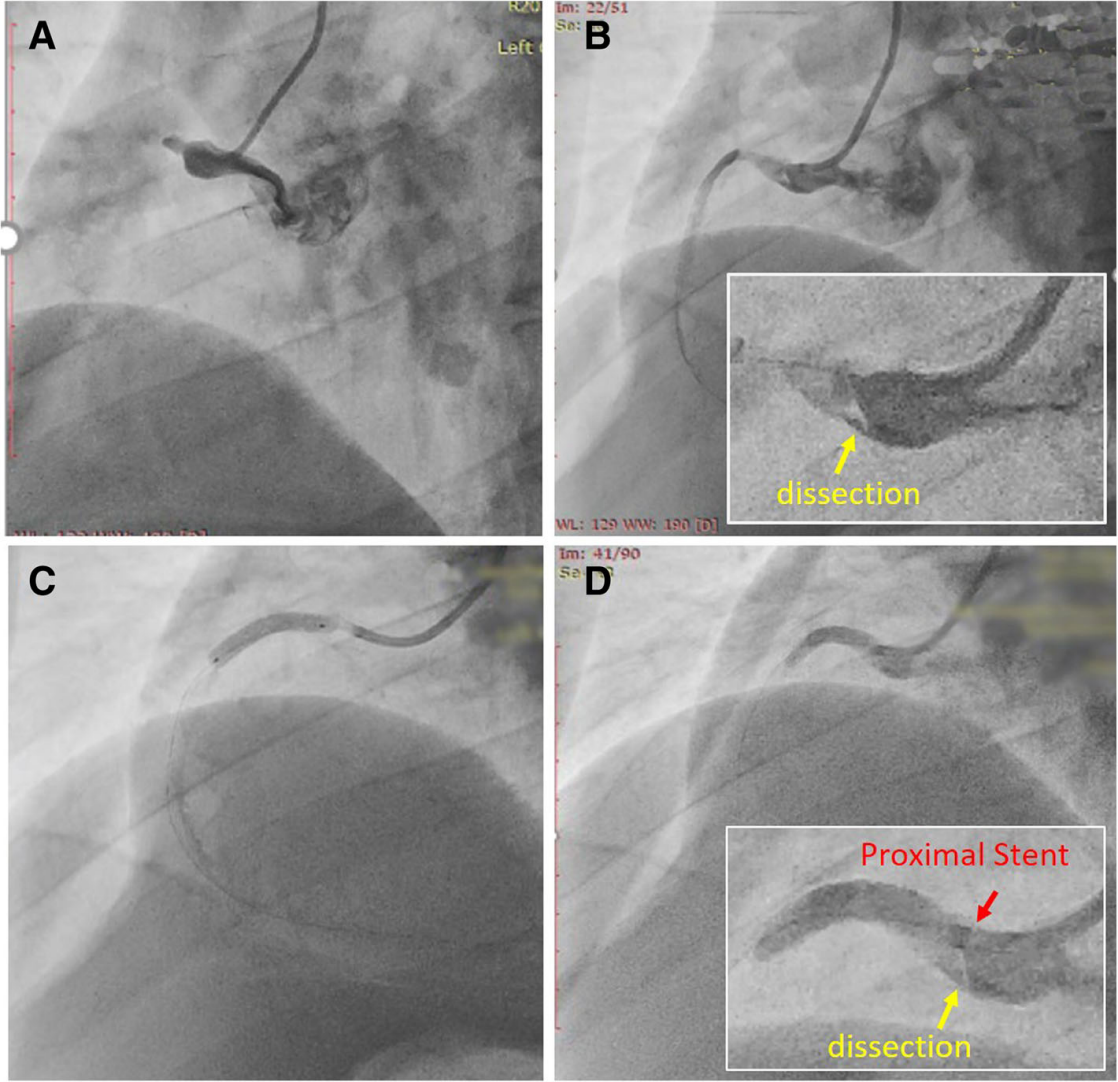
The primary percutaneous coronary artery intervention therapy for acute proximal right coronary artery occlusion in another hospital. **a** Angiography for right coronary artery. **b** Angiography after balloon predilation. **c** Stent implement in false lumen of proximal right coronary artery. **d** Final angiography

A 40-year-old Chinese man had a history of ST-segment elevation inferior myocardial infarction 6 months earlier. He received primary PCI therapy at another hospital. Following predilation with a 2.0 × 20-mm balloon at 12 atm for 6 seconds, a 3.5 × 24 mm sirolimus-eluting stent (EXCEL, JW Medical Systems, Shandong Province, China) was implanted in the lesions of the proximal RCA. However, after stent implantation, coronary angiography (CAG) showed TIMI grade 0 flow in the RCA (Fig. 1). He did not receive further PCI therapy because he had no persistent chest pain at that time. He was prescribed regular dual anti-platelet (PLT) therapy with aspirin and clopidogrel, as well as statin treatment.

Six months later, he was admitted to our hospital for recurrent unstable angina. He denied a history of hypertension, diabetes mellitus, and valvular heart disease. He also denied being allergic to pollen, dust medications, or food, and surgical procedures and injuries. He was married at the age of 23 years and he had one boy and one girl. He received aspirin (0.1 g per day) and clopidogrel (75 mg per day) prior to admission. He had smoked 20 cigarettes per day for 10 years, and had drunk a little amount of alcohol for 20 years.

A physical examination showed: temperature (T) 36.4 °C, pulse (P) 85/minute, respiratory rate (RR) 20/minute, and blood pressure (BP) 130/80 mmHg. He was well developed, moderately nourished, and active. His skin was not stained yellow, with no cyanosis, pigmentation, skin eruption, or spider angioma. There was no pitting edema.

A heart examination revealed no bulge, abnormal impulse, or thrills in precordial area. The border of his heart was normal, and the point of maximum impulse was in his fifth left intercostal space inside the mid clavicular line and it was not diffuse. There was no pericardial friction sound. His heart sounds were strong and there was no splitting. His cardiac rhythm was regular with no pathological murmurs.

A neurological examination showed normal abdominal, bicipital muscular reflex, patellar and heel-tap reflex with Babinski sign (−), Oppenheim sign (−), Gordon sign (−), Chaddock sign (−), Hoffmann sign (−), Kernig sign (−), and Brudzinski sign (−).

An electrocardiogram (ECG; 24 October 2017) in our hospital showed that deep Q waves were present in the II, III, and aVF leads, suggesting old lower wall myocardial infarction. An initial echocardiogram revealed a left ventricular ejection fraction (LVEF) of 41%.

Laboratory findings (25 October 2017) in our hospital showed: red blood cells (RBC) 4.41 × 10^12^/L, white blood cells (WBC) 5.81 × 10^9^/L, N 71.9%, hemoglobin (HGB) 133 g/L, PLT 225 × 10^9^/L, aspartate aminotransferase (AST) 10.5 U/L, alanine aminotransferase (ALT) 11.7 U/L, creatinine (Cr) 7.2 mmol/L, and blood urea nitrogen (BUN) 102 umol/L. Cardiac biomarkers of troponin T (TnT), creatine kinase (CK), and isoenzyme of CK (CK-MB) were negative.

**Fig. 2:**
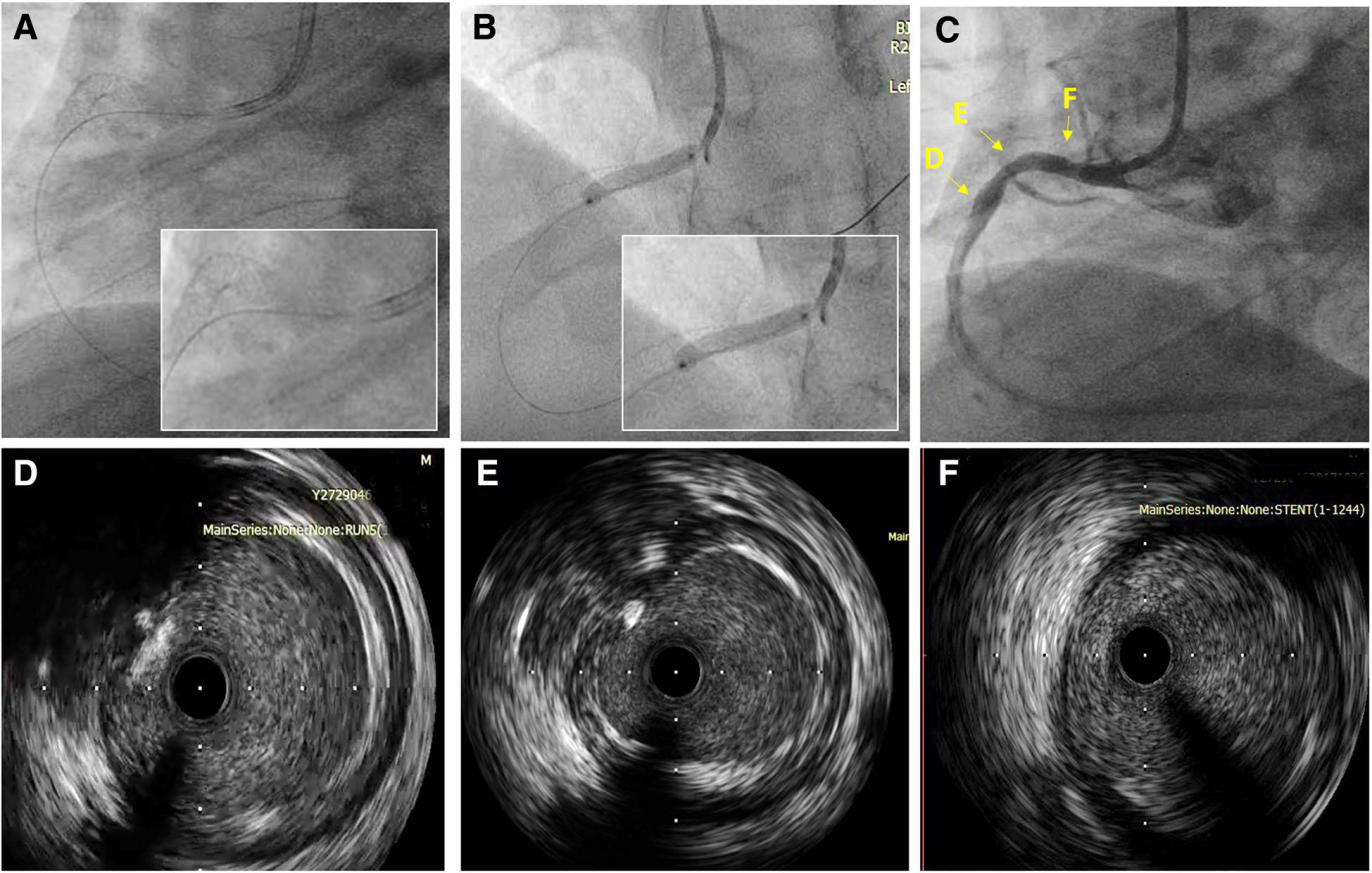
Coronary angiography and intravascular ultrasound. **a** Wire re-entry into true lumen of right coronary artery. **b** Coronary angiography revealed that the wire was in true lumen of right coronary artery. **c** Coronary angiography after true lumen stent implementation. **d**–**f** Intravascular ultrasound confirmed that wire was in the true lumen from distal, mid, and proximal right coronary artery respectively

A repeat CAG showed that although the proximal edge of the previous stent exhibited total occlusion, flow into the distal RCA through another pathway could be seen. The JR4.0 guide catheter was placed immediately outside the ostium of the RCA, and we adjusted the direction of the guide wire to direct it from the ostial true lumen into the distal RCA. Then, IVUS was performed to confirm that the previously deployed stent was in the false lumen, resulting in stent occlusion, and that this guide wire did not go through the struts of the previous proximal stent [2, 3]. A balloon (Sprinter 2.0 × 20 mm, Medtronic, Minneapolis, Minnesota, USA) was then predilated at 14–16 atm to crush the previous stent. Another 4.0 × 20 mm stent (BuMA™, SINOMED, Tianjin, China) was deployed in the true lumen of the proximal RCA to crush the previous stent, followed by postdilation with a 4.0 × 12 mm balloon at 16–18 atm. Blood flow into the RCA finally recovered to TIMI grade III (Fig. 2). Another 12 months of dual anti-PLT therapy was recommended to prevent stent thrombosis and restenosis. Our patient completed his 6-month and 9-month out-patient follow-up visits with no complaints of discomfort (Table 1).

**Table 1:** Timeline

14 April 2017	Chest pain, sweating, and vomiting
14 April 2017	The patient was admitted to a local hospital and received primary percutaneous coronary artery intervention therapy
21 April 2017	Discharged home to take dual anti-platelet and statin treatment
24 October 2017	The patient was admitted to Cardiology department of our hospital for recurrent unstable angina.
24 October 2017	He received percutaneous coronary artery intervention therapy for right coronary artery
30 October 2017	Discharged home. We recommended that the patient take dual anti-platelet and statin treatment for another 12 months.

## Discussion

Stent restenosis or occlusion is a potentially life-threatening complication of PCI that may cause myocardial infarction or heart failure. Here, we report a case of repeat stent implementation to completely rescue occlusion of the RCA by crushing a previous stent in the false lumen [4]. We also described the procedural steps of how to open the false lumen, perform IVUS-guided wire into the true lumen, and how to avoid rewiring the mesh of the previous stent in detail, all these differed from other literature.

There are several points we can learn from this case. First, imaging results from angiography must be read carefully, and confirming the true lumen before PCI is important. Repeat CAG revealed flow into the distal RCA from the ostial true lumen. Then, a JR4.0 guide catheter was placed outside the ostium to allow guide wire entry into the true lumen of the proximal RCA [5]. IVUS proved to be a pivotal tool in confirming false or true lumen, as well as determining favorable proximal site entry points to avoid rewiring the mesh of the previous stent. Furthermore, there are some surgical points that should be considered to avoid wiring the false lumen. Contralateral angiography, microcatheter angiography, and IVUS can be performed to confirm whether the guide wire is in the true lumen. Physicians should not predilate lesions with a balloon or deploy a stent if they are uncertain that the guide wire is in the true lumen. Physicians should also avoid aggressively engaging the ostium of the coronary artery with the guide catheter, and it is safer to place the guide catheter immediately outside the ostium to avoid catheter-induced dissection.

## Conclusion

IVUS proved to be a pivotal tool in confirming false or true lumen, as well as determining favorable proximal site entry points to avoid rewiring the mesh of the previous stent.
